# Synergistic Effect of Network-Based Multicomponent Drugs: An Investigation on the Treatment of Non-Small-Cell Lung Cancer with Compound Liuju Formula

**DOI:** 10.1155/2019/9854047

**Published:** 2019-12-26

**Authors:** Xing Su, Meng Jiang, Yueping Li, Jinglin Zhu, Chunli Zheng, Xuetong Chen, Jun Zhou, Wei Xiao, Yonghua Wang, Yan Li

**Affiliations:** ^1^Key Laboratory of Xinjiang Phytomedicine Resource and Utilization, Ministry of Education, Shihezi University, Shihezi 832002, China; ^2^Key Laboratory of Resource Biology and Biotechnology in Western China, Ministry of Education, School of Life Sciences, Northwest University, Xi'an 710000, China; ^3^Lab of Systems Pharmacology, Center of Bioinformatics, College of Life Science, Northwest A&F University, Yangling, China; ^4^State Key Laboratory of New-tech for Chinese Medicine Pharmaceutical Process, Jiangsu Kanion Parmaceutical Co. Ltd., Lianyungang 222002, China; ^5^Key Laboratory of Industrial Ecology and Environmental Engineering (MOE), Department of Materials Sciences and Chemical Engineering, Dalian University of Technology, Dalian, Liaoning 116024, China

## Abstract

Lung cancer is the most common cause of cancer death with high morbidity and mortality, which non-small-cell lung cancer (NSCLC) accounting for the majority. Traditional Chinese Medicine (TCM) is effective in the treatment of complex diseases, especially cancer. However, TCM is still in the conceptual stage. The interaction between different components remains unknown due to its multicomponent and multitarget characteristics. In this study, compound Liuju formula was taken as an example to isolate compounds with synergistic biological activity through systems pharmacology strategy. Through pharmacokinetic evaluation, 37 potentially active compounds were screened out. Meanwhile, 116 targets of these compounds were obtained by combing with the target prediction model. Through network analysis, we found that multicomponent drugs can present a synergistic effect through regulating inflammatory signaling pathway, invasion pathway, proliferation, and apoptosis pathway. Finally, it was confirmed that the bioactive compounds of compound Liuju formula have not only a killing effect on NSCLC tumor cells but also a synergistic effect on inhibiting the secretion of correlative inflammatory mediators, including TNF-*α* and IL-1*β*. The systems pharmacology method was applied in this study, which provides a new direction for analyzing the mechanism of TCM.

## 1. Introduction

With increasing incidence and mortality, cancer has become the leading cause of death and has caused serious public health problems worldwide. Among them, lung cancer is the most common type of cancer [1, 2]. Non-small-cell lung cancer (NSCLC) accounts for 80–85% of all lung cancers [3]. At present, the main effective methods in reducing the mortality of non-small-cell lung cancer include chemotherapy, radiotherapy, targeted therapy, and surgery [4]. Nonetheless, the overall 5-year survival rate of NSCLC remains low and is only 18% [5], while the drug resistance and side effects are getting more serious [2]. Therefore, there is an urgent need for novel methods for treating NSCLC that are effective and safe.

In recent years, Traditional Chinese Medicine (TCM) is widely used all over the world. TCM has been effective to relieve complex diseases for over 4000 years due to multicomponent, multitarget, and multilevel characteristics [6]. For instance, compound Liuju formula is widely used in the treatment of lung diseases in China. Clinical medicines such as clinical compound Liuju tablets and compound Liuju granules contain leaves of Hanliuye (*Salix matsudana* Koidz., SMK), Yejuhua (*Chrysanthemum indicum* L., CIL), and Baihuasheshecao (*Hedyotis diffusa* Willd, HDW). The SMK was used in the Chinese dictionary [7] as a traditional anti-inflammatory and analgesic [7, 8] medicine. Research has shown that CIL has been used to cure inflammation-related diseases and malignant tumors [9, 10]. It is reported that EEHDW has an effective inhibitory activity on human lung cancer cells by inhibiting cell proliferation and reducing cell activity [11]. TCM had fewer side effects, wide availability and better effect, and can availably improve the life quality of NSCLC patients. However, it is very hard to explain the interaction among the collaborative compositions that we confront a huge complex system for TCM formula.

With the development of analytical tools such as biology network [12], network pharmacology [13], and systems biology [14], the complex and comprehensive mechanism of TCM syndrome differentiation is expected to be quickly and efficiently clarified. In previous research, we have successfully constructed a new systems pharmacology method in order to explore the potential mechanism of Traditional Chinese Medicine. This method combines pharmacokinetics (absorption, distribution, metabolism, excretion, and toxicity (ADME/T) characteristics of drugs), molecular evaluation, target prediction, and pathway analysis to explore the effects of drugs [15, 16], which provides a platform for identifying multiple mechanisms of action of drugs. This platform has successfully developed four herbs, including Radix *Astragali Mongolici*, Radix *Puerariae Lobatae*, Radix *Ophiopogonis Japonici*, and Radix *Salviae miltiorrhiza* [17], that were applied in the comprehensive treatment of cardiovascular disease. systems pharmacology has become a widely used new tool to reveal the mechanism of drug action and drug development.

In our current work, the systems pharmacology method was utilized to study synergic bioactive compounds isolated from the compound Liuju formula treatment of NSCLC. We screened out bioactive ingredients from the constructed compound Liuju formula via ADME by calculating pharmacokinetic properties and evaluating oral bioavailability (OB) and drug-likeness (DL). Then, a comprehensive target prediction method combining biological model and mathematical model was used to predict the homologous target of the selected bioactive ingredients. Next, the obtained target was verified by functional enrichment analysis and target-disease interaction analysis. Finally, the system revealed potential collaborative interactions between bioactive ingredients, active targets, and pathways through systematic pharmacology theory and NSCLC-related signaling pathway evaluation. And, *in vitro* experiments were conducted to further verify the inhibitory effect of potential bioactive ingredients on tumor cells. These results not only provide new treatments for NSCLC but also promote the elucidation and development of the molecular mechanism of TCM.

## 2. Materials and Methods

### 2.1. Construction of Molecular Database and ADME Screening

All compounds of SMK, CIL, and HDW were obtained from our formerly established database named Traditional Chinese Medicine Systems Pharmacology Database (TCMSP, http://lsp.nwu.edu.cn/) [18] and were detected artificially. Because glycosyl molecules are usually hydrolyzed into free glycosides *in vivo*, which can be easily absorbed by intestinal mucosa, we wrote the molecule without glycosyl viscose as _qt [19]. To gain the bioactive molecules, we employed a comprehensive model comprising predict OB (oral bioavailability) and DL (drug-likeness) to appraise pharmacokinetic and pharmaceutical properties.

#### 2.1.1. Oral Bioavailability

Oral bioavailability (OB) refers to the speed and degree of drug absorption into human circulation, which reflects the proportion of drugs in human circulation and plays a critical role in drug screening. In this work, OB value was calculated by an in-house model OBioavail1.1 [18]. And, the threshold of OB value was positioned as 25% by the following conditions: firstly, get as much information as possible from the herbs studied with the fewest compounds. Secondly, the acquired model is correctly interpreted by the reported pharmacological data [20]. In this work, for further analysis, we limited the OB threshold as 25%.

#### 2.1.2. Drug-Likeness

Drug-likeness (DL) refers to the similarity between compounds and known drugs, which is an important factor in determining the success of final clinical trials of drugs. In this work, we utilized the previously developed internal model (Tanimoto coefficient) [20] to predict drug-like properties of expected molecules. The DL appraisal formula is as follows:

(1) $$\begin{array}{c} T\left(A,B\right)=\frac{A\cdot B}{{\left|A\right|}^{2}+{\left|B\right|}^{2}-A\cdot B}. \end{array}$$

Among them, *A* represents the molecular descriptor of herbal compounds, and *B* represents the average molecular properties of all compounds in the database (http://www.drugbank.ca/) [21]. For further study, we defined DL ≥ 0.18 (average of drug library) as the criterion for screening candidate compounds.

In order to obtain the potential bioactive ingredients, the screening standard was defined as OB ≥ 25%, DL ≥ 0.18.

### 2.2. Drug Targeting

In order to structure a direct link between potential bioactive ingredients and targets, we utilized the in-house developed system drug targeting tool (SysDT) [22] and weighted integration similarity (WES) [23] algorithm to predict the target of the compound and to improve the comprehensiveness and accuracy of the target data bank.

Firstly, the weighted ensemble similarity (WES) and systematic drug targeting tool (SysDT) were applied to explore the target information of active compound. As for SysDT, which includes two mathematical tools, Random Forest (RF) and Support Vector Machine (SVM) can determine the interaction between composite targets more completely [22]. There was another computing model, WES, which combines CDK parameters, Dragon parameters, and CDK-Dragon mixing parameters, to predict the direct target of the actual bioactive ingredients [23]. Secondly, the collected protein targets were mapped to the UniProt database (http://www.uniprot.org) for standardization [24]. Finally, the normalized compound targets were mapped to Therapeutic Target Database (TTD, http://database.idrb.cqu.edu.cn/TTD/) [25], Comparative Toxicogenomics Database (CTD, http://ctdbase.org/) [26], Pharmacogenomics Knowledgebase (PharmGKB, https://www.pharmgkb.org/) [27], and Kyoto Encyclopedia of Genes and Genomes (KEGG, http://www.kegg.jp/) [28] to obtain their corresponding diseases and to screen out a relationship between target and disease network.

### 2.3. Gene Ontology (GO) Analysis and Pathway Enrichment

In order to further analyze the specific biological processes and approaches of the potential targets we have obtained, Gene Ontology (GO) enrichment analysis was performed by linking the targets to the KEGG. KEGG is a collection of databases for systematic analysis of gene functions, biological pathways, diseases, drugs, and chemicals [29]. Finally, the pathway and process enrichment analyses were carried out by using Metascape (Metascape, http://metascape.org) [30, 31] software.

### 2.4. Network Construction

In order to visualize the action mechanism of active compounds treating NSCLC and further clarify the relationship between active targets and compounds, we constructed two relational networks: Compound-Target network (C-T network) and Target-Pathway network (T-P network). In these networks, compounds, targets, and pathways were represented by nodes, while the relationship between them was represented by line segments. The degree represents the number of edges associated with a node, and the larger the number, the more node relationships it represents. The topological properties of these networks were analyzed using Cytoscape 3.6.0 [32], which is fashionable bioinformatics software.

### 2.5. Pathway Construction

In terms of pathway, in order to explore the integrative mechanism of action of compound Liuju formula on NSCLC, an integrated pathway related to NSCLC was established based on existing pathological knowledge of NSCLC. Firstly, in order to obtain the basic information of the pathway, we mapped the screened human target proteins to the KEGG database. Secondly, the integrated KEGG pathways of targets with false discovery rated (FDR) less than 0.05 by Fisher's Exact test in the Database for Annotation, Visualization, and Integrated Discovery (DAVID, http://david.abcc.ncifcrf.gov) (evaluated to Fisher's exact test, FDR < 0.05) were inspected [33]. Finally, we manually assembled a relatively complete NSCLC-related pathway to further analyze the molecular action mechanism.

### 2.6. Experimental Detection

#### 2.6.1. Sample Treatment

Chemicals apigenin (B20981, HPLC ≥ 98%), kaempferol (B21126, HPLC ≥ 98%), and ursolic acid (B21403, HPLC ≥ 98%) were purchased from Shanghai Yuanye BioTechnology Co., Ltd., (Shanghai, China), and the concentration of the original solution prepared with dimethyl sulfoxide (DMSO) (American, Sigma) was 100 mmol/L. In order to ensure that the survival of cells was not affected, the final concentration of DMSO should not exceed 0.1%.

#### 2.6.2. Cell Cultures

The murine macrophage line RAW264.7 cell and human NSCLC cell lines H1975 cells were obtained from Cell Resource Center, Shanghai Institutes for Biological Sciences, and CAS. RAW264.7 and H1975 cells were cultured in DMEM and RPMI 1640 (Gibco, USA) medium, respectively. Supplemented with 10% heat inactivated foetal bovine serum (FBS) and antibiotics (100 units/mL penicillin and 100 *μ*g/mL streptomycin). Cells were survived in the incubator of 5% CO_2_ at 37°C. Culture medium was changed every other day.

#### 2.6.3. Establishment of Inflammation Model

RAW264.7 cells were used to construct inflammatory models. Firstly, the cells were cultured in 150 mm Petri dish for 24 hours and treated with drugs for 2 hours. Then, 0.1 *μ*g/mL lipopolysaccharide (LPS) was added to culture for 18 hours. Finally, the cells were collected, and the expression level of inflammatory mediators was detected.

#### 2.6.4. Cell Cytotoxicity Analysis

Determination of cell cytotoxicity was conducted by Cell Counting Kit-8 (CCK-8) assay (Best Bio, Shanghai, China). In brief, H1975 cells were cultured in 96-well plates at a density of 1 × 10^5^ cells/well. After 24-hour training, cells were exposed to different concentrations of apigenin, kaempferol, and ursolic acid. After treatment for 48 h, 10 *μ*L of CCK-8 assay was added to each well, and the cells were hatched for 1–4 h at 37°C and 5% CO2. The absorbance value at 550 nm was surveyed using a microplate reader (Molecular Devices, USA). The cell viability was calculated as: OD of treatment/OD of control × 100%.

#### 2.6.5. Expression Levels of TNF-*α* and IL-1*β*

Enzyme-linked immunosorbent assay (ELISA) kit (R&D Systems, USA) was used to measure the expression of TNF-*α* and IL-1*β*. The cell supernatant after drug treatment was collected according to the protocol of the specification, and 50 *μ*L of the sample was used for detection. The sample concentration was calculated according to the standards provided in the kit.

### 2.7. Statistical Analysis

Variables were analyzed by Student's *t*-test and one-way ANOVA and post hoc analysis of variance (GraphPad Prism version 7). Results were reported as mean values S.E. ^*∗*^*p* < 0.05; ^*∗∗*^*p* < 0.01, and ^*∗∗∗*^*p* < 0.001.

## 3. Results

### 3.1. Active Compound Screening

To screen out the potential bioactive ingredients of SMK, CIL, and HDW, we appraised the ingredients' ADME properties including OB and DL. As shown in Supporting Information [Supplementary-material supplementary-material-1], the results showed that among 156 compounds, 37 compounds reached the standard of OB ≥ 25%, DL ≥ 0.18. It was worth noting that 6 shared compounds met the screening conditions of SMK, CIL, or HDW, such as *β*-sitosterol, apigenin, luteolin, kaempferol, ursolic acid, and sitogluside-qt, indicating that these active compounds may exhibit effective pharmacological effects on NSCLC. Further, in order to verify whether virtual screening results were consistent with NSCLC, we conducted a literature review of the potential components. Many of the 37 active components had been reported of having significant antitumor and anti-inflammatory effects. For example, *β*-sitosterol (MOL004, OB = 36.91%, DL = 0.75) induced G0/G1 cell cycle arrest and inhibited cell proliferation in A549 cells [34]. Studies have shown that ursolic acid (MOL074, OB = 37.73%, DL = O.75) induces apoptosis via activation of caspases and phosphorylation of glycogen synthase kinase 3 beta in ovarian cancer cells [35]. Apigenin (MOL009, OB = 45.09, DL = 0.21) was affirmed to inhibit the migration/invasion of NSCLC cells harboring different EGFR statuses via suppressing the Snail/Slug-mediated EMT [36]. These potential compounds may be key components in the treatment of NSCLC.

### 3.2. Medicine Targeting and Analysis

To explore the target of compounds for NSCLC, we enriched the targets of the compounds. Therefore, we identified 116 targets of these active compounds by means of the WES and SysDT algorithms (as shown in Table 1). The results indicated that most compounds act on more than one target and exhibit multiple pharmacological effects of biologically active molecules. For example, target peroxisome proliferator-activated receptor gamma (PPARG) corresponds to 23 compounds accounting for 62% of the total active compound. Studies have shown that activation of PPARG, *γ* subtype, could cause proliferation inhibition or differentiation of tumor cells [37]. In addition, elevated coexpression of PTGS2 and NOS2 (51% and 48% of the total compounds, respectively) proteins is a strong predictor of poor survival among cancer patients [38]. Hence, these targets involved in the biological processes of NSCLC will be further researched.

### 3.3. Pathway and Process Enrichment Analyses

**Figure 1:**
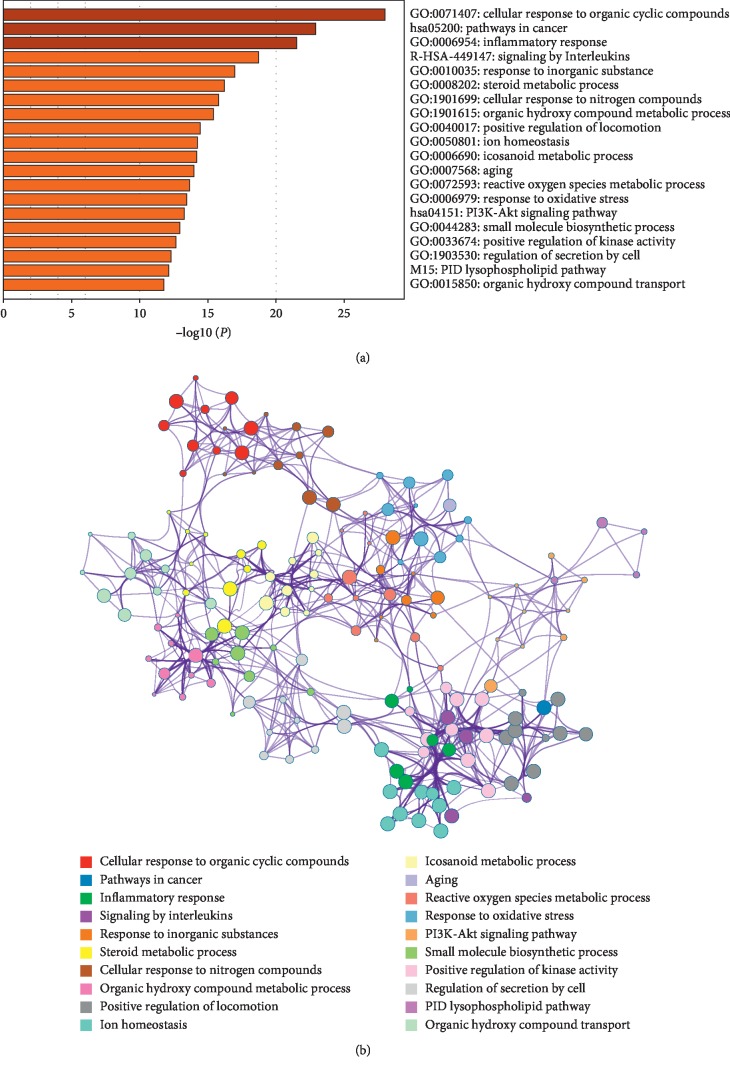
Pathway and process enrichment analyses of the potential targets. (a) Heatmap of enriched terms across input gene lists, colored by *p* values. (b) Network of enriched terms: colored by cluster ID, where nodes that share the same cluster ID are typically close to each other.

Next, we used Metacape software to enrich and analyze the gene ontology (GO) of proteins targeting potential bioactive components to verify whether these proteins are related to NSCLC and to set threshold of *P* value ≤0.05. As shown in Figure 1, we discovered that they all participate in the biological processes such as “cellular response to organic cyclic compound,” “inflammatory response,” “response to inorganic substance,” and “cellular response to nitrogen compound”. Thus, the targets of active molecules we filter from the compound Liuju formula could be regarded as the NSCLC therapeutic targets.

### 3.4. Compound-Target Network Analysis

**Figure 2:**
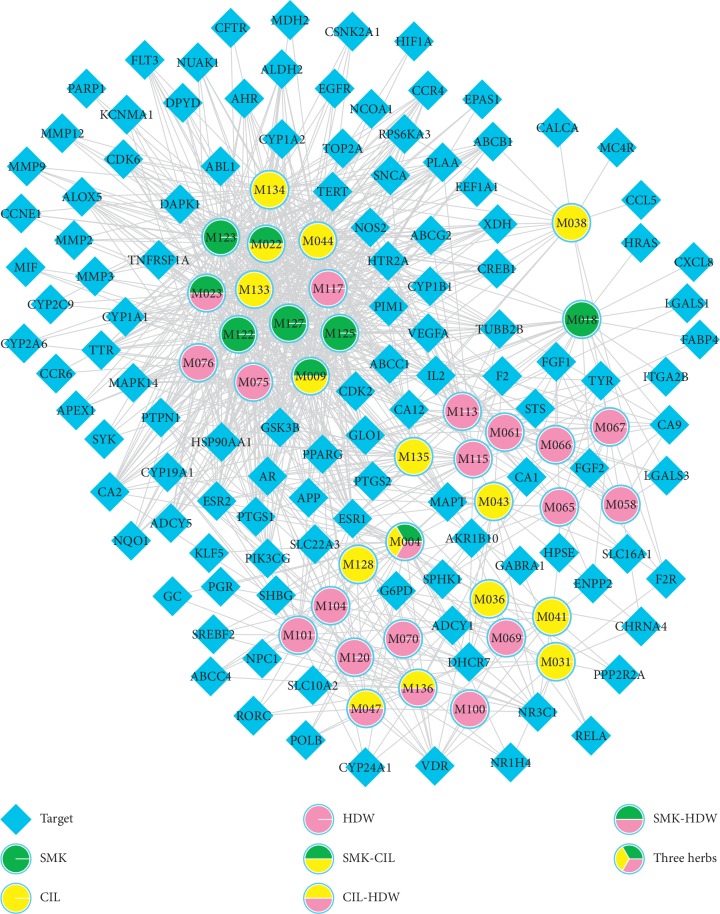
Compound-target network. A compound node and a protein node are linked if the protein is targeted by the corresponding compound. Node size is proportional to its degree.

In this section, we used Cytoscape 3.6.0 to generate the C-T relation network diagram (Figure 2) which contains 950 interactions between 37 molecules and 116 targets to reveal the relationship between the target and the compound more directly. Subsequently, the C-T network topology analysis showed that the average degree of the compound was 31 and the average target degree was 8, respectively. This may mean that each active compound is associated with multiple targets and all play key roles in disease mechanisms.

Notably, apigenin (MOL009, Degree = 51) effectively suppressed lung cancer progression by targeting the CD26-Akt-Snail/Slug signaling pathway [36]. And, the research indicated that kaempferol (MOL023, degree = 48) increased tumor cell killing effect through inhibition of the AKT/PI3K and ERK pathways [39]. Also, ursolic acid (MOL047, Degree = 14) was one of the action components that was present in extracts of CIL and HDW. In recent years, anticancer, anti-inflammation, and regulating immune cell effects of ursolic acid have been discovered [40–42]. Therefore, these three ingredients with a high degree play crucial roles in NSCLC treatment. Significantly, prostaglandin-endoperoxide synthase 2 (PTGS2, Degree = 19) has been found to be highly expressed in many cancer types, and it contributes to tumorigenesis via the inhibition of apoptosis, increased angiogenesis, and invasiveness [43]. All these suggest that compounds probably treat NSCLC by inhibition of tumor cell cycle, anti-inflammation, and inhibiting tumor angiogenesis.

### 3.5. Target-Pathway Network Analysis

**Figure 3:**
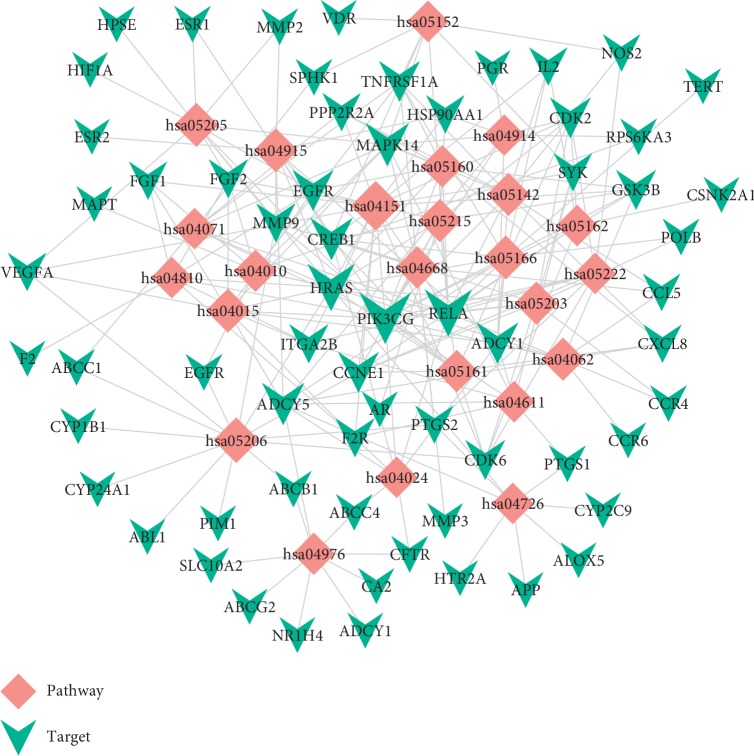
Target-pathway network. The T-P network is built by a target and a pathway if the pathway is lighted at the target. Node size is proportional to its degree.

The results are shown in Table 2; the 24 dramatically enriched pathways (*p* value ≤0.05, multiple targets ≥8) may be the main pathway of action and play a key role in NSCLC disease. As shown in Figure 3, the T-P network includes 88 nodes (64 targets and 24 pathways) and 232 edges. Meanwhile, numerous pathways are regulated by multiple target proteins, which might be the main factor contributing to the anti-NSCLC effect of the herbal formula for NSCLC. pathways in cancer (*p* value = 7.7 *∗* 10^(−12)^, hsa05200, degree = 28), PI3K-Akt signaling pathway (*p* value = 1.0 *∗* 10^(−5)^, hsa04151, degree = 18), MicroRNAs in cancer (*p* value = 1.0 *∗* 10^(−3)^, hsa05206, degree = 13), Proteoglycans in cancer (*p* value = 7.2 *∗* 10^(−4)^, hsa05205, degree = 11), and TNF signaling pathway (*p* value = 1.7 *∗* 10^(−4)^, hsa04668, degree = 9) may be crucial pathways. For instance, the PI3K-AKT pathway may be a key pathway regulating the proliferation and apoptosis of NSCLC cells [44]. Meanwhile, the activation of TNF signaling by inflammatory signaling plays an important role in the development of tumors [45]. Also, heparin sulfate proteoglycans (HSPGs) in the proteoglycans in cancer pathway are key components of the extracellular matrix that mediate cell proliferation, invasion, and cell signaling [46, 47]. Therefore, tumor invasion is an important process of tumor growth and metastasis. So, we infer that the compound activates multiple signaling pathways to inhibit inflammation, enhance immune response, and delay invasion of NSCLC. It is noteworthy that the multitarget enrichment of these pathways provides further theoretical support for the treatment of NSCLC.

### 3.6. NSCLC Disease Pathway Analysis

**Figure 4:**
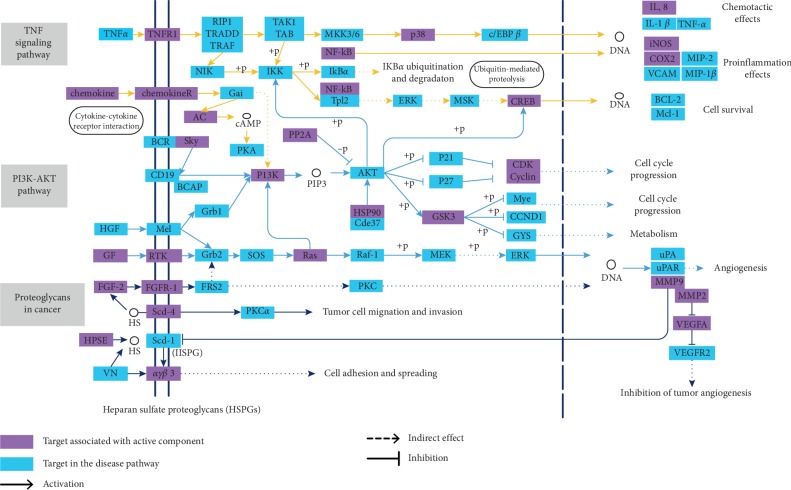
The integrated NSCLC pathway for compound Liuju formula.

To explain the therapeutic mechanism of the active ingredients at the pathway level, based on the target pathway information obtained in the KEGG database, the key pathways obtained from the T-P network analysis were integrated to construct a complete “NSCLC pathway.” As shown in Figure 4, the “NSCLC pathway” includes three signaling pathways, hsa04151: PI3K-Akt signaling pathway, hsa05205: polysaccharide in cancer, and hsa04668: TNF signaling pathway. The integrated pathway reflects multiple modules such as cell proliferation, apoptosis, angiogenesis, and migration.

#### 3.6.1. Cell Cycle Progression Module

PI3K-AKT pathway and TNF signaling pathway are involved in regulating the cell cycle progress. As we mentioned in Figure 4, apigenin (MOL009) was observed to affect cyclin-dependent kinase 2 (CDK2). It plays a key role in cell cycle progression which could accelerate the transition from G1 to S phase, and the dysregulation of CDK2 is closely related to many cancers [48]. Besides, luteolin (MOL022) was predicted to impact the heat shock protein 90 (HSP90), which regulates DNA methyltransferase transcription and silences tumor suppressor and DNA repair gene methylation in tumor development, growth, and therapeutic response plays an important role [49]. Above results indicate that the compound can effectively regulate the expression of PI3K, CDK/cyclin, GSK3, and other genes, thereby activating the pathway and regulating the cell cycle progression.

#### 3.6.2. Inflammation Effects Module

Inflammatory mediators and inflammatory cells are important components of tumor microenvironment and play an important role in the occurrence, development, and metastasis of tumor [50]. Hence, anti-inflammatory is an integral strategy for the compound treatment. As an important indicator of inflammation, the transcription factor p65 (NF-κB) reduces the expression of the downstream protein prostaglandin G/H synthase 2 (COX-2) and nitric oxide synthase (iNOS) by activated luteolin (MOL022) [51]. Studies have shown that COX2 may be affected by systemic inflammation, and the prognostic impact of COX2 expression depends on tumor characteristics [52]. Also, iNOS is the main mediator of inflammation, and iNOS can enhance inflammation and plays an important role in apoptosis [53]. Therefore, the analysis showed that the compound alleviated the symptoms of inflammatory disorders in patients with NSCLC by regulating the anti-inflammatory activities of NF-κB, cox-2, and iNOS.

#### 3.6.3. Invasion Module

Invasion and metastasis of tumor cells are critical to the development of tumors and exacerbate the progression of tumors. In the “NSCLC pathway” shown in Figure 4, the polysaccharide in cancer pathway was involved in regulating the invasion and metastasis progress. Research have shown that tumor cells migration was associated with an increase of *αγβ*3 (MOL022, luteolin) integrin proteasome degradation [54, 55]. Meanwhile, FGF2, also known as basic fibroblast growth factor (bFGF) and FGF-*β*, is a growth factor and signaling protein encoded by the FGF2 gene. It was involved in a variety of biological processes, including cell growth, tissue repair, angiogenesis, tumor growth, and invasion [56, 57]. In addition, VEGFA was modulated by apigenin (MOL009) and luteolin (MOL022). It has been reported in literature that VEGFA has many functions such as increasing vascular permeability, inducing angiogenesis, angiogenesis and endothelial cell growth, promoting cell migration, and inhibiting apoptosis [58–60]. Thus, these above analyses show that compound may treat NSCLC by regulating the angiogenesis, migration, and invasion.

### 3.7. *In Vitro* Experimental Detection

#### 3.7.1. Cell Cytotoxicity Assay

**Figure 5:**
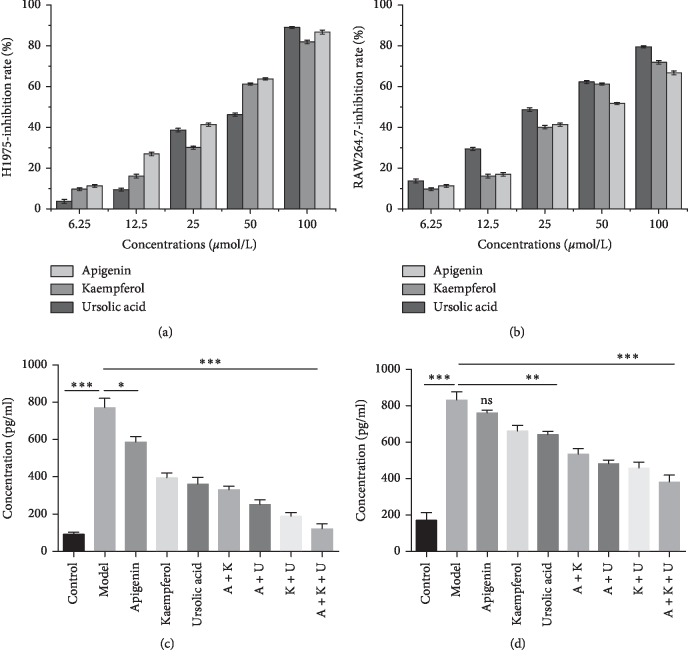
Effect of compound Liuju formula active component at the cellular level. (a, b) Cellular cytotoxicity assay. The *x*-axis shows the drugs concentrations. The *y*-axis shows the inhibition rate of drugs on cells. (c, d) Effect of apigenin, kaempferol, and ursolic acid on LPS-induced production of inflammatory mediators. Expression levels TNF-*α* and IL-1*β* in RAW264.7 cells: experience group cells treated with different dose medicines and analyzed by ELISA. A + K: apigenin and kaempferol; A + U: apigenin and ursolic acid; K + U: kaempferol and ursolic acid; A + K + U: apigenin, kaempferol, and ursolic acid. Data are presented as the mean ± SD (*n* = 3). ^*∗*^*p* < 0.05, ^*∗∗*^*p* < 0.01, and ^*∗∗∗*^*p* < 0.001.

In this section, we used H1975 cell line to verify the compound efficacy. We selected three highly active multitarget components apigenin (MOL009), kaempferol (MOL023) and ursolic acid (MOL047) common to three plants described above for further verification. We used different concentrations of medicine concentration to detect the cytotoxicity of apigenin, kaempferol, and ursolic acid on H1975 and RAW264.7 cell and explored its inhibition rate. The results of cellular cytotoxicity assay (Figures 5(a) and 5(b)) showed that different concentrations of active ingredients significantly inhibited the growth of H1975 and RAW264.7 cells. These results demonstrated that apigenin, kaempferol, and ursolic acid had obvious proliferation inhibition activity on cancer cells.

#### 3.7.2. Expression Levels of TNF-*α* and IL-1*β*

To further confirm the anti-inflammatory activity of apigenin, kaempferol, and ursolic acid, we used RAW264.7 macrophages which were treated by LPS, with or without apigenin, kaempferol, and ursolic acid. Based on previous toxicity results, we chose a dose of 20 *μ*M for further test. Therefore, we examined TNF-*α* and IL-1*β* by ELISA, whose results demonstrated that the apigenin, kaempferol, and ursolic acid can downregulate the levels of TNF-*α*. The combination of drugs is more effective, especially in the three coadministered groups (Figures 5(c) and 5(d)). It is worth noting that the expression of IL-1*β* was not as obvious as TNF-*α*, and the apigenin group was not different from the model group. However, compared with the model group, the expression level of the combined group was significantly reduced. In summary, the results of two groups showed that the combined group was better than the single drug group, and the combination of three drugs was the most obvious one with significant differences.

## 4. Discussion

With increasing incidence and mortality, lung cancer has become the most common cancer and the leading cause of cancer death [61]. In addition, high metastatic rate of lung cancer and its poor prognosis leading to the search for antilung cancer drugs has become an important issue to be solved.

Hence, in this study, we chose compound Liuju formula, a clinically used Chinese medicine compound, as an example to interpret the combination effect of Traditional Chinese Medicine treatment. In order to further reveal the potential action mechanism of active compounds in Traditional Chinese Medicine formulas, we proposed a systematic pharmacological approach to gain a deeper understanding of the synergistic pharmacological mechanisms of compound Liuju formula. Firstly, based on the evaluation method, 37 active ingredients were obtained, and 116 potential disease-related targets were predicted. The results showed that the compound Liuju formula has the characteristics of multicomponent and multitarget synergistic treatment. Then, active compounds and C-T analysis showed that several active compounds in the compound Liuju formula are essential for the treatment of NSCLC, including apigenin, kaempferol, and ursolic acid. Moreover, some targets such as CDK2, COX2, iNOS, and VEGF have anti-inflammatory, antimigration, and antiproliferation effects on NSCLC. In addition, the result of pathway and process enrichment analyses, T-P analyses, and the integrated “NSCLC pathway” suggest that compound Liuju formula mainly treats NSCLC by regulating cellular process, inflammatory response, migration, and invasion. Finally, we have shown that apigenin, kaempferol, ursolic acid has obvious anti-proliferative effect on lung cancer cells by *in vitro* cytotoxicity test results. Furthermore, we determined by ELISA kit that apigenin, kaempferol, and ursolic acid have significant anti-inflammatory effects, especially in the combination treatment group, confirming the synergistic effect between apigenin, kaempferol, and ursolic acid. The formula has a promoting effect on the regulation of tumor inflammatory microenvironment and has a potential research value in the treatment of NSCLC.

In summary, the systems pharmacology method reveals the characteristics of compound Liuju formula with multicomponent Chinese medicine treatment and multitarget effective treatment. And, this strategy provides a potential method for the rational discovery of new medicines.

However, the current methods of systems pharmacology are still in the early stage of development, and the content of the platform needs to be further enriched. Some models, such as ADME screening, need to be optimized, and some database bases should be expanded and updated. And, developing new algorithms, adding more drug-like properties, and improving screening accuracy could enrich the content of the platform. Moreover, the inhibitory effect on the lung cancer of compound Liuju formula was investigated by us in the study, and then we can carry out systems pharmacology prediction on other diseases and other malignant cancers in in-depth exploration. A more comprehensive therapeutic effect of compound Liuju formula would be developed and may contribute new strategies to cancer therapy.

## Figures and Tables

**Table 1:** The information of disease-related targets.

UniProt-ID	Protein names	Gene names	Degree
P03372	Estrogen receptor	ESR1	25
P04150	Glucocorticoid receptor	NR3C1	13
P05067	Amyloid-beta A4 protein	APP	15
P06401	Progesterone receptor	PGR	4
P06746	DNA polymerase beta	POLB	4
P10275	Androgen receptor	AR	27
P10636	Microtubule-associated protein tau	MAPT	13
P11413	Glucose-6-phosphate 1-dehydrogenase	G6PD	13
P11473	Vitamin D3 receptor	VDR	13
P37231	Peroxisome proliferator-activated receptor gamma	PPARG	23
Q08828	Adenylate cyclase type 1	ADCY1	12
Q13887	Krueppel-like factor 5	KLF5	1
Q9NYA1	Sphingosine kinase 1	SPHK1	3
Q9UBM7	7-dehydrocholesterol reductase	DHCR7	14
P00915	Carbonic anhydrase 1	CA1	6
P00918	Carbonic anhydrase 2	CA2	17
P04798	Cytochrome P450 1A1	CYP19A1	11
Q07973	1,25-dihydroxyvitamin D(3) 24-hydroxylase, mitochondrial	CYP24A1	5
Q92731	Estrogen receptor beta	ESR2	18
O15439	Multidrug resistance-associated protein 4	ABCC4	4
O15118	NPC intracellular cholesterol transporter 1	NPC1	4
Q96RI1	Bile acid receptor	NR1H4	4
Q12908	Ileal sodium/bile acid cotransporter	SLC10A2	6
P51449	Nuclear receptor ROR-gamma	RORC	4
O75751	Solute carrier family 22 member 3	SLC22A3	5
P04278	Sex hormone-binding globulin	SHBG	5
Q12772	Sterol regulatory element-binding protein 2	SREBF2	4
P02774	Vitamin D-binding protein	GC	2
O95622	Adenylate cyclase type 5	ADCY5	13
P04798	Cytochrome P450 1A1	CYP1A1	12
P05091	Aldehyde dehydrogenase, mitochondrial	ALDH2	11
P05177	Cytochrome P450 1A2	CYP1A2	10
P07900	Heat shock protein HSP 90-alpha	HSP90AA1	16
P09917	Arachidonate 5-lipoxygenase	ALOX5	13
P11309	Serine/threonine-protein kinase pim-1	PIM1	15
P18031	Tyrosine-protein phosphatase non-receptor type 1	PTPN1	18
P19438	Tumor necrosis factor receptor superfamily member 1A	TNFRSF1A	9
P23219	Prostaglandin G/H synthase 1	PTGS1	14
P24941	Cyclin-dependent kinase 2	CDK2	14
P33527	Multidrug resistance-associated protein 1	ABCC1	14
P35228	Nitric oxide synthase, inducible	NOS2	18
P35354	Prostaglandin G/H synthase 2	PTGS2	19
P36888	Receptor-type tyrosine-protein kinase FLT3	FLT3	7
P47989	Xanthine dehydrogenase/oxidase (includes xanthine dehydrogenase)	XDH	12
P48736	Phosphatidylinositol 4,5-bisphosphate 3-kinase catalytic subunit gamma isoform	PIK3CG	9
P49841	Glycogen synthase kinase-3 beta	GSK3B	15
P68400	Casein kinase II subunit alpha	CSNK2A1	4
Q00534	Cyclin-dependent kinase 6	CDK6	12
Q12791	Calcium-activated potassium channel subunit alpha-1	KCNMA1	4
Q12882	Dihydropyrimidine dehydrogenase [NADP(+)]	DPYD	4
Q16539	Mitogen-activated protein kinase 14	MAPK14	15
Q16678	Cytochrome P450 1B1	CYP1B1	14
Q9BVA1	Tubulin beta-2B chain	TUBB2B	15
Q9UNQ0	ATP-binding cassette subfamily G member 2	ABCG2	13
Q9Y263	Phospholipase A-2-activating protein	PLAA	12
P00519	Tyrosine-protein kinase ABL1	ABL1	7
P35869	Aryl hydrocarbon receptor	AHR	8
O43570	Carbonic anhydrase 12	CA12	18
P51679	C-C chemokine receptor type 4	CCR4	10
P13569	Cystic fibrosis transmembrane conductance regulator	CFTR	2
P16220	Cyclic AMP-responsive element-binding protein 1	CREB1	12
P53355	Death-associated protein kinase 1	DAPK1	11
P60568	Interleukin-2	IL2	18
P51812	Ribosomal protein S6 kinase alpha-3	RPS6KA3	11
P43405	Tyrosine-protein kinase SYK	SYK	4
Q04760	Lactoylglutathione lyase	GLO1	15
P08183	Multidrug resistance protein 1	ABCB1	12
P14174	Macrophage migration inhibitory factor	MIF	3
P15559	NAD(P)H dehydrogenase [quinone] 1	NQO1	6
O14746	Telomerase reverse transcriptase	TERT	11
P02766	Transthyretin	TTR	12
P15692	Vascular endothelial growth factor A	VEGFA	17
P00734	Prothrombin	F2	9
P08514	Integrin alpha-IIb	ITGA2B	2
P08842	Steryl-sulfatase	STS	3
P15090	Fatty acid-binding protein, adipocyte	FABP4	2
P28223	5-hydroxytryptamine receptor 2A	HTR2A	8
Q16790	Carbonic anhydrase 9	CA9	8
P05230	Fibroblast growth factor 1	FGF1	7
P09038	Fibroblast growth factor 2	FGF2	7
P10145	Interleukin-8	CXCL8	1
P09382	Galectin-1	LGALS1	2
P17931	Galectin-3	LGALS3	4
P01112	GTPase HRas	HRAS	1
P14679	Tyrosinase	TYR	9
P08253	72 kDa type IV collagenase	MMP2	4
P08254	Stromelysin-1	MMP3	4
P09874	Poly [ADP-ribose] polymerase 1	PARP1	2
P14780	Matrix metalloproteinase-9	MMP9	4
P24864	G1/S-specific cyclin-E1	CCNE1	4
P39900	Macrophage metalloelastase	MMP12	4
O60285	NUAK family SNF1-like kinase 1	NUAK1	6
P37840	Alpha-synuclein	SNCA	5
P11712	Cytochrome P450 2C9	CYP2C9	3
P51684	C-C chemokine receptor type 6	CCR6	2
P27695	DNA-(apurinic or apyrimidinic site) lyase	APEX1	4
P11388	DNA topoisomerase 2-alpha	TOP2A	5
O60218	Aldo-keto reductase family 1 member B10	AKR1B10	9
P25116	Proteinase-activated receptor 1	F2R	5
P43681	Neuronal acetylcholine receptor subunit alpha-4	CHRNA4	4
P63151	Serine/threonine-protein phosphatase 2A 55 kDa regulatory subunit B alpha isoform	PPP2R2A	3
Q04206	Transcription factor p65	RELA	3
P14867	Gamma-aminobutyric acid receptor subunit alpha-1	GABRA1	3
P06881	Calcitonin gene-related peptide 1	CALCA	1
P13501	C-C motif chemokine 5	CCL5	1
P32245	Melanocortin receptor 4	MC4R	1
P68104	Elongation factor 1-alpha 1	EEF1A1	3
Q13822	Ectonucleotide pyrophosphatase/phosphodiesterase family member 2	ENPP2	3
Q9Y251	Heparanase	HPSE	3
P53985	Monocarboxylate transporter 1	SLC16A1	2
P11509	Cytochrome P450 2A6	CYP2A6	3
Q15788	Nuclear receptor coactivator 1	NCOA1	1
Q99814	Endothelial PAS domain-containing protein 1	EPAS1	3
P00533	Epidermal growth factor receptor	EGFR	4
Q16665	Hypoxia-inducible factor 1-alpha	HIF1A	2
P40926	Malate dehydrogenase, mitochondrial	MDH2	2

**Table 2:** The information of disease-related pathways.

Term	Pathways	Degree
hsa05200	Pathways in cancer	28
hsa04151	PI3K-AKT signaling pathway	18
hsa05206	MicroRNAs in cancer	13
hsa04915	Estrogen signaling pathway	11
hsa05205	Proteoglycans in cancer	11
hsa04015	Rap1 signaling pathway	11
hsa05166	HTLV-I infection	11
hsa05215	Prostate cancer	10
hsa05142	Chagas disease (American trypanosomiasis)	10
hsa04062	Chemokine signaling pathway	10
hsa05203	Viral carcinogenesis	10
hsa04976	Bile secretion	9
hsa04668	TNF signaling pathway	9
hsa05160	Hepatitis C	9
hsa05161	Hepatitis B	9
hsa04010	MAPK signaling pathway	9
hsa05222	Small-cell lung cancer	8
hsa04914	Progesterone-mediated oocyte maturation	8
hsa04726	Serotonergic synapse	8
hsa04071	Sphingolipid signaling pathway	8
hsa04611	Platelet activation	8
hsa05162	Measles	8
hsa05152	Tuberculosis	8
hsa04024	cAMP signaling pathway	8
hsa04810	Regulation of actin cytoskeleton	8

## Data Availability

The data such as active constituent ADME parameters, disease-related targets, and disease-related pathways to support the findings of this study are included within the article. The data such as compound structure, pathway and process enrichment analyses, compound-target network, Target-Pathway network, and the integrated NSCLC pathway used to support the findings of this study are included within the supplementary information file. The cellular level effect data used to support the findings of this study are available from the corresponding author upon request because part of the data for this result is included in the mentor's fund project, and it is temporarily not suitable for disclosure.

## Supplementary Materials

Supplementary Materials Supplementary Figure 1: Graphical Abstract. Table 1: Active constituents and their corresponding ADME parameters.
