# Supplementary material for: Synergistic Effect of Network-Based Multicomponent Drugs: An Investigation on the Treatment of Non-Small-Cell Lung Cancer with Compound Liuju Formula
Source: Evid Based Complement Alternat Med. 2019 Dec 26;2019:9854047. doi: 10.1155/2019/9854047 (PMC6948348; doi:10.1155/2019/9854047)
Supplement: Supplementary Materials — Supplementary Figure 1: Graphical Abstract. Table 1: Active constituents and their corresponding ADME parameters. [file 9854047.f1.docx]

**Graphical Abstract**


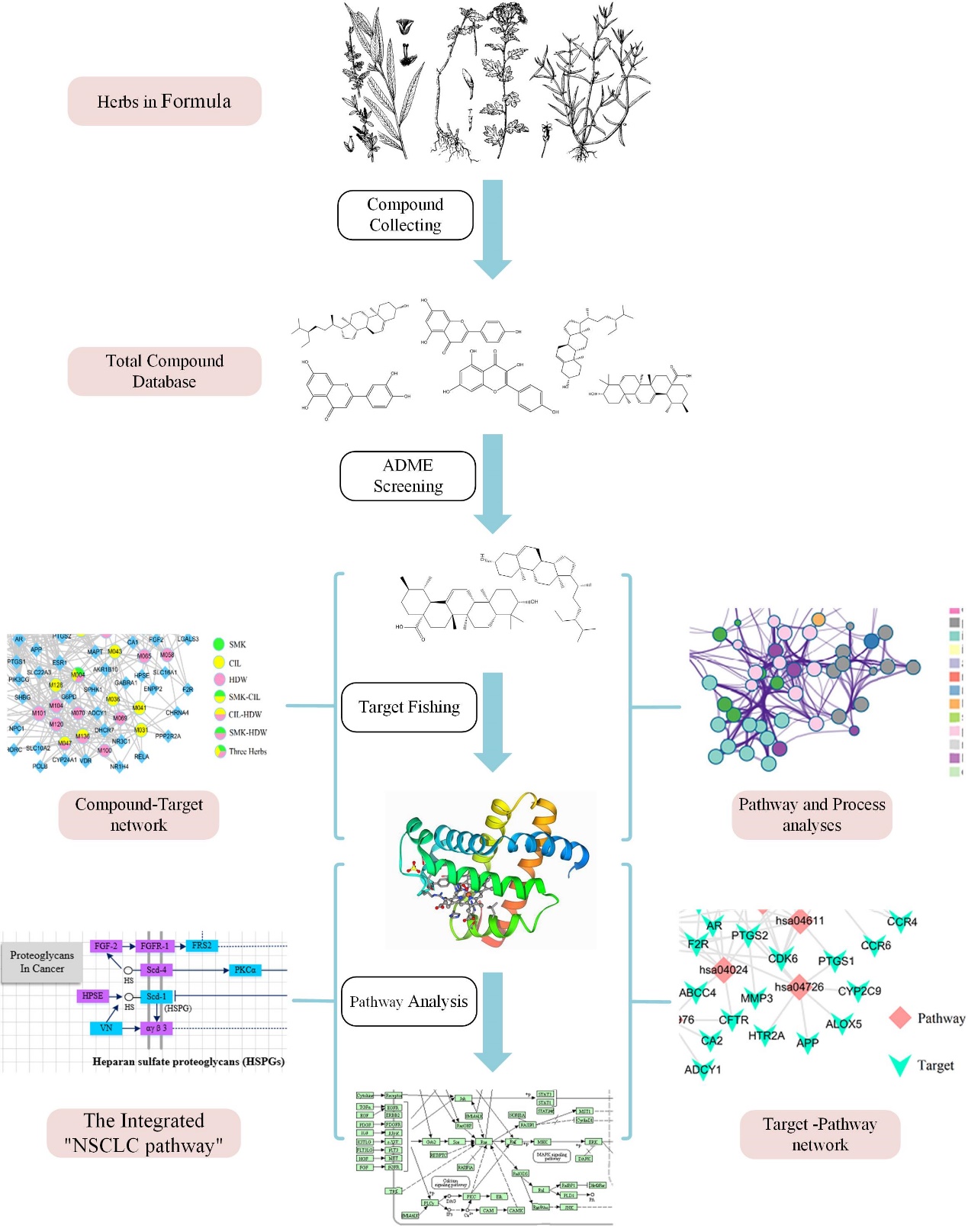


As shown in the figure, we introduce the method of Systems Pharmacology to resolve the potential mechanism of herbal medicines in the treatment of NSCLC. First, based on ADME evaluation system, we screened out the active compounds from the constructed Total Compound Database by calculating the pharmacokinetic properties and evaluating their oral bioavailability (OB) and drug-likeness (DL). Then, the homologous targets of these active compounds were predicted based on the comprehensive target prediction method based on the combination of biological model and mathematical model. Next, the obtained targets were verified by functional enrichment analysis and target disease interaction analysis. Finally, the network pharmacology and NSCLC-related signaling pathways were evaluated, and the potential interactions among active components, active targets and pathways were systematically revealed.

**Table 1 Active constituents and their corresponding ADME parameters**.

| MOL-ID | Compounds | MW | CID | OB | DL | HL | CACO2 | Degree | Structure |
| --- | --- | --- | --- | --- | --- | --- | --- | --- | --- |
| M004 | β-sitosterol | 414.79 | 222284 | 36.91 | 0.75 | Short | 1.32663 | 28 |  |
| M009 | apigenin | 270.25 | 5280443 | 45.09 | 0.21 | Long | 0.41493 | 51 |  |
| M018 | tremuloidin | 390.42 | 3083619 | 40.89 | 0.44 | Short | -0.63516 | 25 |  |
| M022 | luteolin | 286.25 | 5280445 | 26.37 | 0.25 | Long | 0.20456 | 53 |  |
| M023 | kaempferol | 286.25 | 5280863 | 69.61 | 0.24 | Long | 0.24956 | 48 |  |
| M031 | handelin | 552.72 | 90474153 | 34.24 | 0.31 | Long | -0.1856 | 12 |  |
| M036 | arteglasin B | 320.37 | 169495 | 77.51 | 0.37 | Long | -0.062 | 10 |  |
| M038 | linarin | 592.6 | 5317025 | 39.84 | 0.71 | Long | -1.643 | 17 |  |
| M041 | chrysanthemaxanthin | 584.96 | 5376313 | 58.90 | 0.56 | Long | 0.66664 | 11 |  |
| M043 | cumambrin A | 306.39 | 174867 | 77.86 | 0.27 | Long | 0.32099 | 13 |  |
| M044 | acacetin | 284.28 | 5280442 | 37.35 | 0.24 | Long | 0.64258 | 53 |  |
| M047 | ursolic acid | 456.78 | 64945 | 37.73 | 0.75 | Long | 0.55549 | 14 |  |
| M058 | asperuloside | 414.4 | 84298 | 26.43 | 0.71 | Short | -1.47008 | 10 |  |
| M061 | paederoside | 446.47 | 442432 | 57.19 | 0.76 | Short | -1.55035 | 10 |  |
| M065 | (E)-6-O-p-coumaroyl scandoside methyl ester | 550.56 | 44584784 | 27.12 | 0.81 | Short | -1.922 | 14 |  |
| M066 | (Z)-6-O-p-coumaroyl scandoside methyl ester | 550.56 | 44584783 | 27.12 | 0.81 | Short | -2.16007 | 9 |  |
| M067 | (E)-6-O-feruloyl scandoside methyl ester | 580.59 | 46225505 | 34.71 | 0.76 | Short | -1.88799 | 13 |  |
| M069 | isoarborinol | 426.8 | 12305182 | 39.51 | 0.77 | Short | 1.38099 | 8 |  |
| M070 | lupenylacetate | 468.84 | 12900957 | 42.35 | 0.76 | Long | 1.39681 | 7 |  |
| M075 | 2-methyl-3-methoxy anthoxy anthraquinone | 284.28 | 91038674 | 71.39 | 0.27 | Long | 0.35921 | 20 |  |
| M076 | 2-hydroxy-3-methyl anthraquinone | 238.25 | 10889963 | 26.03 | 0.18 | Short | 0.51523 | 27 |  |
| M100 | squalene | 410.8 | 638072 | 33.55 | 0.43 | Long | 1.96958 | 2 |  |
| M101 | stigmasterol | 412.77 | 5280794 | 43.83 | 0.76 | Short | 1.31024 | 21 |  |
| M104 | 6-Hydroxystigmasta-4,22-dien-3-one | 426.75 | 71307323 | 45.31 | 0.79 | Short | 0.78751 | 16 |  |
| M113 | Coniferin | 342.38 | 5280372 | 29.18 | 0.27 | Short | -1.17154 | 25 |  |
| M115 | ( + ) -neoolivil | 376.44 | 9976812 | 60.28 | 0.39 | Short | -0.25174 | 22 |  |
| M117 | isoscutellarein | 286.25 | 5281665 | 37.81 | 0.24 | Long | 0.32325 | 39 |  |
| M120 | gypsogenic acid | 486.76 | 15560324 | 32.33 | 0.72 | Short | -0.08524 | 11 |  |
| M122 | apigenin-7-O-β-D-glucopyranoside-qt | 270.25 | 5280443 | 57.23 | 0.21 | Long | 0.43264 | 51 |  |
| M123 | luteolin-7-O-β-D-glucopyranoside-qt | 286.25 | 5280445 | 29.64 | 0.25 | Long | 0.22424 | 53 |  |
| M125 | geranin-7-O-β-D-glucoside/Diosmetin 7-O-glucoside-qt | 300.28 | 5281612 | 56.97 | 0.27 | Long | 0.45916 | 45 |  |
| M127 | rutin-qt | 302.25 | 5280343 | 43.44 | 0.28 | Short | 0.0293 | 56 |  |
| M128 | corchorusin D-qt | 456.78 | 5321059 | 25.69 | 0.66 | Short | 0.57256 | 9 |  |
| M133 | linarin-qt | 284.28 | 5280442 | 37.24 | 0.24 | Long | 0.65025 | 52 |  |
| M134 | quercimeritrin-qt | 302.25 | 5280343 | 45.05 | 0.28 | Short | 0.04951 | 56 |  |
| M135 | cyanidin 3-glucoside chloride-qt | 287.26 | 128861 | 37.94 | 0.24 | Short | 0.06857 | 15 |  |
| M136 | sitogluside-qt | 414.79 | 222284 | 36.91 | 0.75 | Short | 1.43887 | 24 |  |

Note: "Long" in the Half-life (HL) parameter means value ≥ 4 h, and "short" means value < 4 h

As shown in Table 1, we collected the active components of SMK, CIL and HDW and their corresponding ADME parameters, including MW, CID, OB, DL, HL, CACO2, Degree, and structure, through the TCMSP Database and literature research.
